# Temporal and Spatial Stability of Ammonia-Oxidizing Archaea and Bacteria in Aquarium Biofilters

**DOI:** 10.1371/journal.pone.0113515

**Published:** 2014-12-05

**Authors:** Samik Bagchi, Siegfried E. Vlaeminck, Laura A. Sauder, Mariela Mosquera, Josh D. Neufeld, Nico Boon

**Affiliations:** 1 Laboratory of Microbial Ecology and Technology (LabMET), Ghent University, Coupure Links 653, 9000 Gent, Belgium; 2 Department of Biology, University of Waterloo, Waterloo, Ontario, Canada; University of Ottawa, Canada

## Abstract

Nitrifying biofilters are used in aquaria and aquaculture systems to prevent accumulation of ammonia by promoting rapid conversion to nitrate via nitrite. Ammonia-oxidizing archaea (AOA), as opposed to ammonia-oxidizing bacteria (AOB), were recently identified as the dominant ammonia oxidizers in most freshwater aquaria. This study investigated biofilms from fixed-bed aquarium biofilters to assess the temporal and spatial dynamics of AOA and AOB abundance and diversity. Over a period of four months, ammonia-oxidizing microorganisms from six freshwater and one marine aquarium were investigated at 4–5 time points. Nitrogen balances for three freshwater aquaria showed that active nitrification by aquarium biofilters accounted for ≥81–86% of total nitrogen conversion in the aquaria. Quantitative PCR (qPCR) for bacterial and thaumarchaeal ammonia monooxygenase (*amoA*) genes demonstrated that AOA were numerically dominant over AOB in all six freshwater aquaria tested, and contributed all detectable *amoA* genes in three aquarium biofilters. In the marine aquarium, however, AOB outnumbered AOA by three to five orders of magnitude based on *amoA* gene abundances. A comparison of AOA abundance in three carrier materials (fine sponge, rough sponge and sintered glass or ceramic rings) of two three-media freshwater biofilters revealed preferential growth of AOA on fine sponge. Denaturing gel gradient electrophoresis (DGGE) of thaumarchaeal 16S rRNA genes indicated that community composition within a given biofilter was stable across media types. In addition, DGGE of all aquarium biofilters revealed low AOA diversity, with few bands, which were stable over time. Nonmetric multidimensional scaling (NMDS) based on denaturing gradient gel electrophoresis (DGGE) fingerprints of thaumarchaeal 16S rRNA genes placed freshwater and marine aquaria communities in separate clusters. These results indicate that AOA are the dominant ammonia-oxidizing microorganisms in freshwater aquarium biofilters, and that AOA community composition within a given aquarium is stable over time and across biofilter support material types.

## Introduction

Ammonia (NH_3_) is toxic to fish at concentrations exceeding 0.1 mg NH_3_-N L^−1^ [1], which can be particularly problematic for confined ecosystems such as ornamental aquaria, ponds, and recirculating aquaculture systems. Ammonia sources include direct excretion through fish gills as well as ammonification of organic nitrogenous compounds (e.g., uneaten feed and faeces). Therefore, nitrifying biofilters are utilized in engineered aquatic systems to promote the conversion of NH_3_ to nitrate (NO_3_). For decades, ammonia-oxidizing bacteria (AOB) belonging to the β- and γ-Proteobacteria were thought to be solely responsible for nitrification in aquarium biofilters and other natural and engineered environments [2]. This idea was challenged by the isolation of *Nitrosopumilus maritimus*, an autotrophic archaeon that gains energy through the oxidation of ammonia to nitrite [3]. In fact, ammonia-oxidizing archaea (AOA) may have been first organisms capable of generating energy through the oxidation of ammonia [4], and although initially considered to be deep-branching Crenarchaeota, several unique characteristics led to the reclassification of AOA to a new phylum, the Thaumarchaeota [5], [6]. Metagenomic surveys and targeted retrieval of thaumarchaeal 16S rRNA and *amoA* genes demonstrate the ubiquity and abundance of AOA in natural ecosystems [7]–[12] and engineered environments, such as wastewater treatment plants [13]–[15], groundwater distribution systems [16] and freshwater aquarium biofilters [17], [18]. High substrate affinity of AOA (e.g. *K*_m_  = 1.86–9.66 µg N L^−1^ total ammonia for *N. maritimus*) may enable adaptation to limited nutrient conditions [19], [20] and explain the presence of AOA in natural oligotrophic environments with low ammonia concentrations [21], [22].

Despite the isolation of *N. maritimus* from marine aquarium sediment, the ecology of ammonia-oxidizing communities in aquarium biofilters is unclear, and the possible roles of AOA and AOB in aquarium nitrification have been addressed in few studies. Urakawa and co-workers [18] examined ammonia oxidizers in three marine aquaria operated at different temperatures (6, 19 and 20°C), and found AOA dominance in all systems. In contrast, Foesel and colleagues [23] found that AOB dominated in a marine aquaculture biofilter (8.9% of the total bacterial population), with a negligible proportion of Archaea (<0.1%). Similarly, Pedersen and colleagues [24] detected Archaea (<1% of the microbial cell counts) in a freshwater aquaculture biofilter, but detected AOB in higher abundances. Recently, Brown *et al.* [25] showed the dominance of group I.1a AOA over AOB in a marine recirculating aquaculture system. Another large-scale sampling of aquarium biofilters showed AOA dominance in 23 of 27 freshwater systems and in 5 of 8 marine systems and that AOA communities were distinct between freshwater and marine aquaria [17]. For one freshwater aquarium, four samples collected over two years showed a persistent AOA dominance [17]. However, all reports of AOA and AOB abundance in aquarium biofilters focused on one-time grab samples. Although such initial studies indicate AOA dominance in aquarium biofilters, studies based on one-time sampling fail to determine the stability of aquarium biofilter ammonia-oxidizing communities. Previous studies on engineered systems showed that nitrification activity is directly related to the stability of nitrifying community over time [26], and stability of nitrifying communities in aquarium biofilters is important for effective removal of ammonia. We hypothesized that AOA dominate freshwater aquarium biofilters and remain stable over time and in different biofilter compartments.

In order to investigate temporal stability of aquarium biofilter communities, we investigated six freshwater aquaria and one marine aquarium over a period of four months. This study assessed the temporal variation of AOA and AOB abundance and thaumarchaeal community composition. In addition, we assessed the abundance and diversity of *Thaumarchaeota* in two freshwater aquaria that contained biofilters comprised of three distinct support media materials to assess whether support material type influences AOA abundance or community composition. Finally, we estimated the contribution of biofilter nitrification to the overall nitrogen balance.

## Material and Methods

### Maintenance and sampling of aquaria

For the temporal variation test, six freshwater aquaria (F1–F6) and one marine aquarium (M) were sampled over a period of 79–113 days from different locations in Gent, Belgium (Table 1). Aquaria F1, F2, and M were household aquaria and permission for sampling was given by Erik Lievens, Kikvorsstraat 1069, Gent. Aquarium F3 was from the Department of Applied Ecology and Environmental Biology, Ghent University, and permission was granted by Prof. Peter Goethals, Ghent University. Aquaria F4–F6 were in-house aquaria of LabMET, Ghent University, and the authors who are affiliated with this lab required no permission. We confirmed that the field studies did not involve endangered or protected species.

**Table 1:** Details of all studied aquaria with associated water quality data.

Type	Aquarium code	Sampling days	Sampling codes	Aquarium water volume (L)	Water change regimen	pH	Dissolved O_2_ (mg O_2_ L^−1^)	Temperature (°C)	TAN (mg N L^−1^)	PO_4_^3-^ (mg P L^−1^)	SO_4_^2-^ (mg L^−1^)	Fish type	Live plants
**Freshwater (no N budget)**	F1	1, 35, 55, 76, 104	F1_1, F1_2, F1_3, F1_4, F1_5	450	10% biweekly	6.0±0.3	6.7±0.5	24.7±0.5	0.45±0.40	1.1±1.0	106±27	27 mixed tropical fish	Yes
	F2	1, 40, 61, 89	F2_1, F2_2, F2_3, F2_4	1050	Unspecified	7.3±0.5	6.8±0.3	24.7±0.5	0.20±0.11	1.9±0.7	221±25	22 African cichlids	Yes
	F3	1, 28, 57, 85, 113	F3_1, F3_2t, F3_2b, F3_3, F3_4t, F3_4b, F3_5t, F3_5b	175	15% weekly	8.0±0.2	9.1±0.1	18.5±1.0	0.26±0.39	0.2±0.1	80±20	*Asellus aquaticus* (crustacean)	No
**Freshwater (N budget)**	F4	1, 22, 50, 79	F4_1t, F4_1b, F4_2t, F4_2b, F4_3, F4_4	200	25% weekly	7.6±0.1	6.5±0.3	24.2±1.3	0.18±0.20	1.9±1.0	83±18	10 African cichlids, 1 *Hypostomus plecostomus*	No
	F5 (temporal test)	1, 22, 50, 79	F5_1t, F5_1b, F5_2, F5_3t, F5_3b, F5_4	80	25% weekly	8.0±0.2	7.6±0.2	23.2±1.5	0.16±0.10	0.9±0.8	83±20	4 *Carassius auratus* (goldfish)	No
	*F5 (spatial test)	1', 20', 40'	F5s_1t, F5s_1m, F5s_1b, F5s_2t, F5s_2m, F5s_2b, F5s_3t, F5s_3m, F5s_3b	80	25% weekly	7.7±0.1	7.6±0.2	23.0±0.3	<0.1	N.A.	N.A.	4 *Carassius auratus* (goldfish)	No
	F6 (temporal test)	1, 29, 57, 86	F6_1, F6_2, F6_3, F6_4	40	25% weekly	7.9±0.5	6.5±0.5	28.1±0.5	0.17±0.12	1.3±0.6	113±77	4 *Xiphophorus helleri,* 2 *Gymnocorymbus ternetzi,* 1 *Hypostomus plecostomus*	No
	*F6 (spatial test)	1', 20', 40'	F6s_1t, F6s_1m, F6s_1b, F6s_2t, F6s_2m, F6s_2b, F6s_3t, F6s_3m, F6s_3b	40	25% weekly	8.0±0.1	6.7±0.1	26.5±0.3	<0.1	N.A.	N.A.	4 *Xiphophorus helleri,* 2 *Gymnocorymbus ternetzi,* 1 *Hypostomus plecostomus*	No
**Marine**	M	1, 14, 63, 77, 105	M_1, M_2, M_3, M_4, M_5	550	10% weekly	7.8±0.2	7.4±0.3	24.3±1.4	0.30±0.04	0.2±0.0	209±96	17 mixed marine fish and reefs	No

b, m, t: biofilter bottom, middle and top compartment, respectively. N.A.: not available

* spatial test was conducted 6 months after temporal study.

Freshwater aquaria F1–F3 and the marine aquarium M were categorized as aquaria with an unspecified nitrogen budget. Conversely, the nitrogen budgets of aquaria F4–F6 were controlled with defined amounts of fish feed (Table 2) and ∼25% of the water volume replaced weekly. Water samples were collected on a weekly basis from these aquaria and filter biomass was collected once per month, during biofilter rinsing, resulting in four or five total time points, depending on the aquarium (Table 1). Water samples were filtered over a 0.45 µm membrane and stored at −20°C until nitrogen analyses were performed. The effect of support media on AOA abundance and diversity was assessed using Aquaria F5 and F6, which were outfitted with biofilters that had three-media designs, including fine sponge (∼0.85 mm pore size), rough sponge (∼1.27 mm pore size) and sintered glass or ceramic rings as support materials. Aquarium F5 had an existing three-media biofilter system and aquarium F6 had its existing single-media filter replaced a by three-media biofilter following the temporal tests (Table 2). This three-media filter was inoculated with biomass from the original F6 filter, and allowed to stabilize for 6 months. After this period, water and biomass samples were taken from F5 and F6 every three weeks, for a total of three time points (Table 2). For both aquaria, ∼25% of the water was replaced by tap water on a weekly basis, consistent with the temporal test.

**Table 2:** Feeding and filter details for the three freshwater aquaria with controlled nitrogen budgets.

Aquarium code	Fish wet weight (g)	Biofilter volume (L)	Biofilter hydraulic residence time (s)	Upflow velocity (m/h)	Carrier material	Fish feed	Protein content (%)	Scenario 1 (days 0-21)		Scenario 2 (days 22-end)	
								Feed dose (mg d^−1^)	Loading rate (mg N L^−1 ^d^−1^) min - max	Feed dose (mg d^−1^)	Loading rate (mg N L^−1 ^d^−1^) min - max
F4	239	15	56	27	t: fine sponge b:rough sponge	Tetra Cichlid flakes	48	200	0.3–0.73	1000	3.2–3.7
F5	190	5	36	9.6	t: sintered glass m: sponge b:ceramic rings	Tetra Goldfish flakes	42	200	1.6–1.9	500	4.1–4.8
F6 (temporal test)*	49	0.13	1.2	13	sponge	Vitakraft Vita Flake-Mix, Sera Viformo tablets	47, 45.7	80, 30	28–34	200, 40	84–103
F6 (spatial test)	49	6.1	31	19	t: fine sponge m:rough sponge b:ceramic rings	Vitakraft Vita Flake-Mix, Sera Viformo tablets	N.A.	NA	NA	NA	NA

Feeding doses were for cycles of five days on, two days off. The minimum biofilter loading rates are based on the measured nitrate production rates, while the maximum rates assume that all fed nitrogen is nitrified.

b, m, t: biofilter bottom, middle and top compartment, respectively. N.A.: not applicable.

* For F6 (temporal test), two different fish feed and their corresponding protein content and feed dose are shown by comma-separated text.

For sampling biomass from F3–F6 and M aquaria, filter media were gently rinsed in tap water and the supernatant was collected in a sterile container. After decanting the supernatant, settled biomass was collected in 50-mL sterile tubes. At low levels of residual chlorine (∼0.06 mg Cl L^−1^), no harmful biological effect was expected by rinsing with tap water. For sampling biofilter fine sponge material from F1 and F2, small slices of biofilter sponge material were excised for sampling. The biomass samples were stored at −20°C until DNA extraction was performed.

### Aquarium nitrogen balance

The nitrogen budget was controlled in aquaria F4–F6 to estimate the nitrification rates of these selected biofilters. Because ammonium never accumulated above 0.5 mg NH_4_^+^-N L^−1^ and nitrite was always below detection limits, nitrogen balances were based solely on nitrate. Between water exchanges, the nitrate concentration was monitored. The nitrate increase was compared to the input of nitrogen in the aquarium in the form of fish feed, calculated from feed addition rates and the manufacturer's nutritional information (Table 2). The feeding rate was increased from day 22 onwards. With the weekly water exchange (∼25% of the total water volume), a considerable amount of nitrate was removed from the aquaria, and replaced by tap water containing 3.0±0.8 mg NO_3_^—^N L^−1^. A cumulative approach of concentration increase over time was employed to cancel out small deviations in the weekly concentration measurements, as the nitrate concentration increases on a weekly basis were relatively low (∼2 mg NO_3_^—^N L^−1^).

### DNA extraction and quantitative PCR

Genomic DNA was extracted from the sponge filters and collected biomass using the FastDNA SPIN Kit (Qbiogene, Carlsbad, CA) according to the manufacturer's protocol, using one milliliter of mixed biomass. Genomic DNA extracts were visualized by standard gel electrophoresis and the measured using a Nanodrop 1000 spectrophotometer (Thermo Scientific, Wilmington, DE, USA).

The qPCR was performed on the StepOne Plus system (Applied Biosystems, Foster City, CA). All amplifications were performed in triplicate with a reaction volume of 25 µL, containing 12.5 µL of Power SYBR Green PCR master mix (Applied Biosystems), 5 pmol of each primer, 5 µg 2% (w/v) of bovine serum albumin (Hoffmann-La Roche Ltd., Basel, Switzerland) and 5 µL of diluted (10^−1^ and 10^−2^) sample at concentration of 1–10 ng DNA per reaction. Archaeal and bacterial *amoA* genes were amplified using CrenamoA23f/CrenamoA616r and amoA-1F/amoA-2R, respectively [7], [27]. For archaeal *amoA* genes, PCR conditions were 40 cycles of 94°C for 1 min, 56°C for 1 min and 60°C for 2 min, followed by a fluorometric plate read. For bacterial *amoA*, qPCR conditions were: 40 cycles of 94°C for 30 s, 55°C for 30 s and 60°C for 45 s, followed by a fluoremetric plate read. For all amplification reactions, melting curves from 65 to 95°C were performed after each run with an incremental increase in temperature of 0.5°C. Two different dilutions (10^−1^ and 10^−2^) of each sample were amplified to validate the quantification by differences in cycle threshold (C_t_) values for each dilution.

PCR amplicons of *Candidatus* Nitrosophaera gargensis were used as a standard for the AOA *amoA* gene [28], and DNA from an oxygen-limited autotrophic nitrification/denitrification (OLAND) reactor biomass was used as a standard for the AOB *amoA* gene [29]. The PCR amplicons were first cloned into the pCR 2.1 TOPO cloning vector (Invitrogen Carlsbad, CA) according to the manufacturer's protocol. Plasmids from transformed cells were extracted by the PureYield Plasmid Miniprep System (Promega, Madison, WI). Because vector and PCR insert sizes were known, copy numbers were calculated from the concentration of extracted plasmid DNA. Standard curves were constructed using serial dilutions of standard template DNA plotted against the C_t_ values for each dilution. Most slopes ranged between −3.4 to −3.6 and coefficients of determination (*R* ^2^) ranged from 0.988 to 0.999. Melting curves calculated for each target sequence showed single peaks and all PCR products were verified by standard gel electrophoresis on a 1% agarose gel. Starting DNA copy numbers for each sample were calculated from the linear regression equation of each standard curve. Detection limits were 213 and 1000 gene copies per reaction for bacterial and thaumarchaeal *amoA* genes (corresponding to 4.2 and 9.7 gene copies ng^−1^ DNA), respectively. The relative abundance of thaumarchaeal and bacterial *amoA* genes was calculated assuming 1 and 2.5 *amoA* gene copies per AOA and AOB cell, respectively [30].

### Denaturing gel gradient electrophoresis (DGGE) and band sequencing

A previous study demonstrated that DGGE from *amoA* and thaumarchaeal 16S rRNA genes produce similar patterns [15], but 16S rRNA gene amplification may reduce the possibility of primer mismatches to known AOA community members. Thus, DGGE fingerprinting for thaumarchaeal 16S rRNA genes was performed as described previously [7]. Briefly, primers 771F and 957R-GC generated 16S rRNA amplicons, which were run on 8% acrylamide gels with a 35%–70% denaturing gradient. Gels were run at 60°C and 85 V for 15 h. The DGGE system used was a DGGEK-2401 (C.B.S. Scientific, Del Mar, CA). Gels were stained with SYBR green I (Invitrogen) for 1 h, and then scanned using the PharosFX system (Bio-Rad, Hercules, CA). Fingerprints were normalized and aligned with GelCompar II (Applied Maths, Austin, TX, USA) and an unweighted pair group method with arithmetic mean (UPGMA) dendrogram was constructed based on Pearson correlations of background-subtracted densitometric curves. Selected individual DGGE bands were excised, PCR amplified, and sequenced. Amplified bands were run on a second gel to ensure both band purity and that the sequenced band corresponded to the original fingerprint. These sequences have been deposited in GenBank with accession numbers KJ557114–KJ557132.

### Ordination plot and statistical analysis

An operational taxonomic unit (OTU) table was generated based on normalized DGGE band intensities using GelCompar II. The Bray-Curtis distance matrix was generated from the OTU table and plotted into a nonmetric multidimensional scaling (NMDS) ordination using the statistical software PC-ORD (version 6). We performed multivariate response permutation procedures (MRPP), a class of multivariate permutation tests of group differences, to observe community differences among aquaria. Multivariate Dispersion indices (MVDISP) and pair-wise analysis of similarity (ANOSIM) analysis on the generated Bray-Curtis distance was calculated using the statistical software PRIMER 6 (version 6.1.13) and PERMANOVA+ add on (version 1.0.3) to calculate the degree of dispersion from the initial sampling and similarity in community structure, respectively. In these analyses, a greater value indicates a greater dissimilarity between samples while a value of zero indicates no significant difference.

### Chemical analyses

Total ammonia nitrogen (TAN; NH_4_^+^-N and NH_3_-N) was measured using Nessler's reagent [31] for freshwater samples and using the NANOCOLOR Ammonium 3 test (Macherey-Nagel, Duran, Germany) for saltwater samples. Additional water chemistry measurements, including NO_2_^-^, NO_3_^-^, SO_4_^2-^, and PO_4_^3-^, were determined using a compact ion chromatograph equipped with a conductivity detector (Metrohm, Zofingen, Switzerland). Separation and elution of anions were carried out on a Metrosep A Supp 5 column (flow 0.7 mL min^−1^; sample loop 20 µL), utilizing carbonate/bicarbonate eluent and auto suppressor technology. Dissolved oxygen (DO) concentrations and temperature were measured with an HQ30d DO meter (Hach Lange, Düsseldorf, Germany). In aquaria F3–F6, the pH was measured with a C532 meter (Consort, Turnhout, Belgium). For aquaria F1, F2, and M, the pH was measured using pH test strips (Merck, Darmstadt, Germany). The experimental results were statistically analyzed using Minitab 15 (Minitab Inc., State College, PA).

## Results

### Aquarium samples

To understand AOA and AOB temporal abundance, samples from six freshwater and one marine aquarium biofilter were collected over a period of 79–113 days. Overall, the tested aquaria had a wide pH range (6.0–8.0), temperature (19–28°C), dissolved oxygen concentrations (6.5–9.0 mg O_2_ L^−1^) and varied in their fish and crustacean compositions (Table 1). The selected aquaria had various commercial biofilters (e.g., three-media to single-media) and ranged in size from 40 to 1050 L, reflecting conditions common to most residential or recreational aquaria. The aquaria contained a variety of fish including mixed tropical, African cichlids, and goldfish; aquarium F3 contained crustaceans, but no fish. Marine aquarium M had live corals in addition to marine fish.

### Temporal AOA and AOB abundance

**Figure 1:**
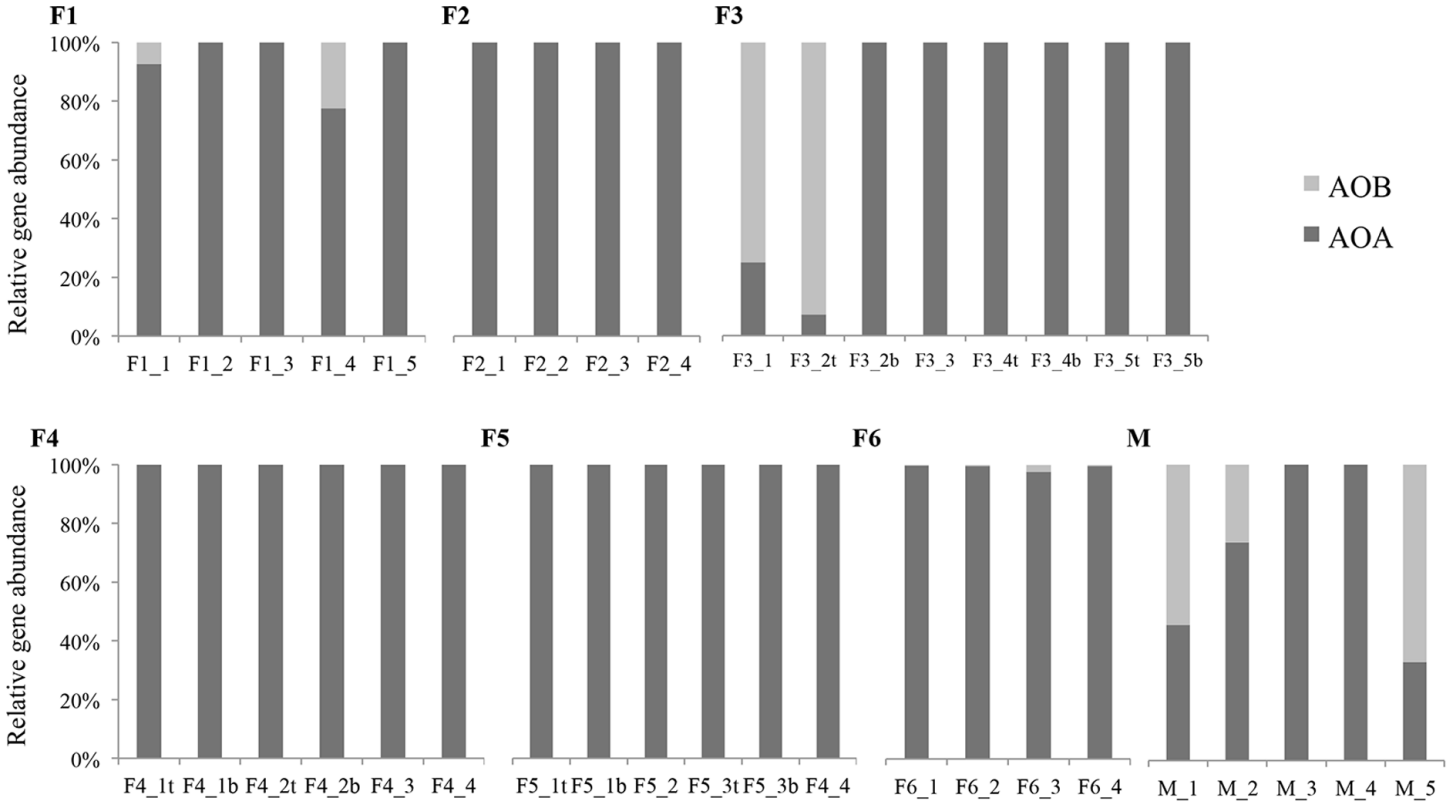
Relative *amoA* gene abundance of Thaumarchaea and Bacteria in sampled freshwater (F1–F6) and marine (M) aquaria, assuming 1 and 2.5 copies of *amoA* gene per thaumarchaeal and bacterial cell, respectively.

The qPCR results demonstrated that thaumarchaeal *amoA* genes were dominant in all freshwater biofilters (Figure 1, Table S1). Thaumarchaeal *amoA* genes represented the entire detected *amoA* signal for three out of the six freshwater aquaria (F2, F4 and F5) at all time points over the four-month period. Although AOB *amoA* genes were detected in the other three aquaria, their abundance was low and variable. In the case of F1, AOB contributed <20% of the total *amoA* genes detected. In aquarium F6, AOB accounted for <5% of the ammonia-oxidizing community (Figure 1). AOA outnumbered AOB by 200- to 400-fold in F6. The only exception was aquarium F3, in which AOB *amoA* genes were present in relatively high proportions (2.1–8.8×10^3^ copies ng^−1^ DNA), outnumbering AOA by 7- to 30-fold during the initial period of one month, then remained undetectable for subsequent sampling, even after repeated qPCR analyses (Figure 1, Table S1).

For the marine aquarium biofilter, *amoA* genes of both AOA and AOB were detected at all time points. Although bacterial *amoA* gene copy numbers were three to five orders of magnitude higher for the initial and final sampling, AOA *amoA* genes were the only detected *amoA* genes for the other sampled time points (Figure 1). On average, AOB *amoA* genes accounted for ∼40% of the total marine *amoA* gene signal. The absence of AOB *amoA* genes during days 63 and 72 was unexpected; repeated qPCR analyses also failed to amplify any AOB *amoA* genes from those samples. Overall, the AOA copy number varied over time in all biofilters, yet AOA dominance over AOB was consistent in sampled freshwater aquaria.

### Temporal AOA diversity

**Figure 2:**
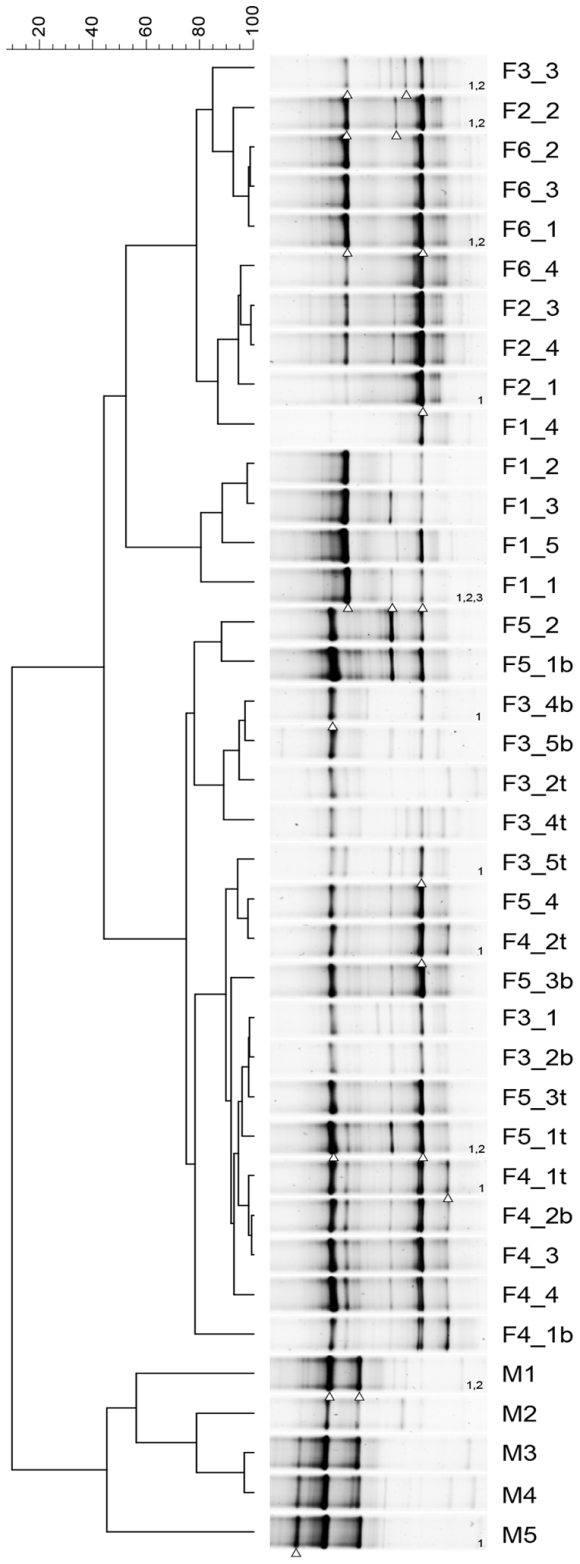
Denaturing gradient gel electrophoresis of thaumarchaeal 16S rRNA gene amplicons from freshwater (F1–F6) and marine (M) aquarium, with an unweighted pair group method with arithmetic mean (UPGMA) dendrogram representing distances based on Pearson correlations of fingerprint densitometric curves. Fingerprints have been normalized and aligned. Bands chosen for sequencing are indicated with triangles and numbering on the right side of lanes.

**Figure 3:**
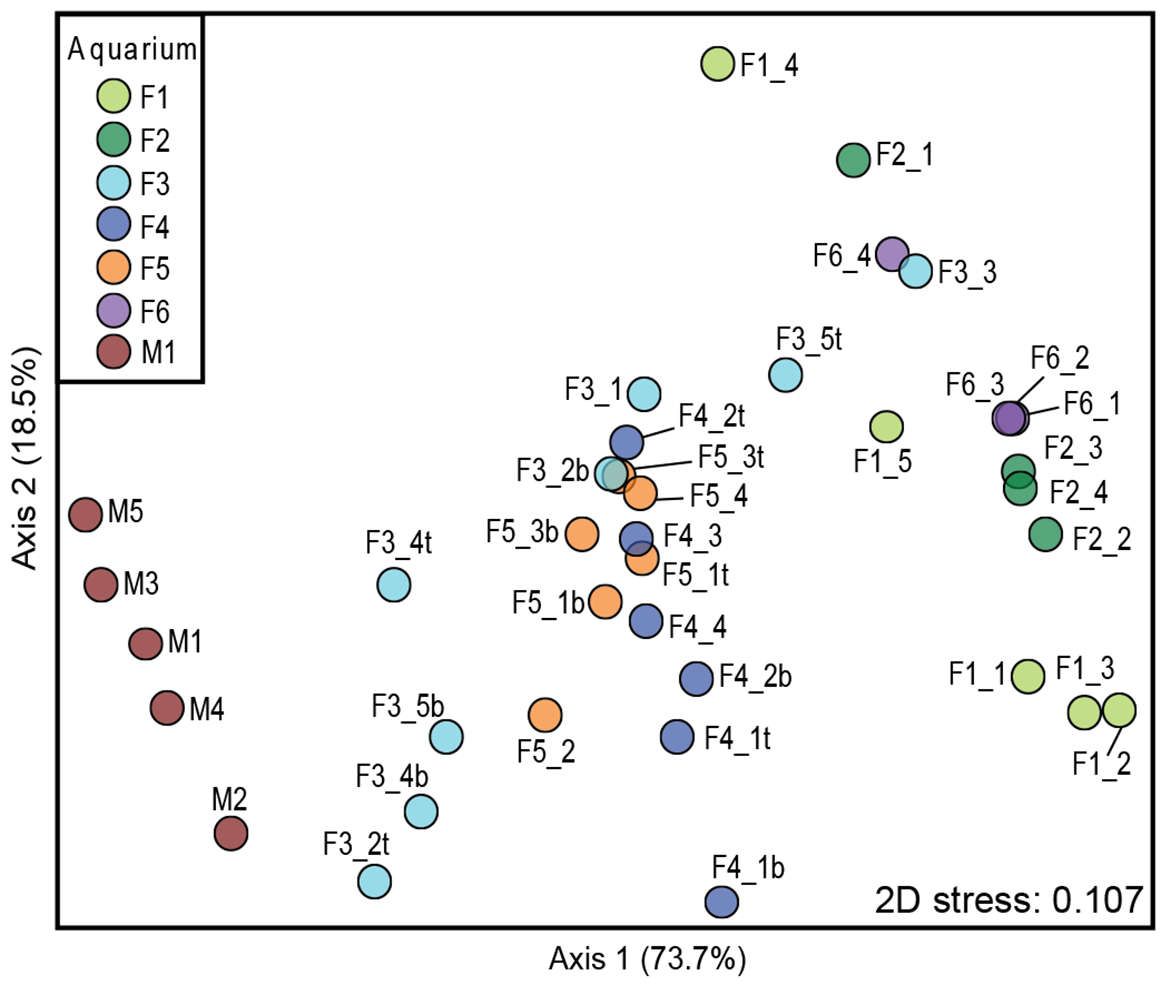
Nonmetric multidimensional scaling (NMDS) ordination of thaumarchaeal 16S rRNA gene DGGE fingerprint. The two-dimensional stress value for the NMDS was 0.107 based on Bray Curtis distance. Coefficients of determination (*R* ^2^) on each axis represent correlations between ordination distances and the corresponding distance matrix.

To characterize AOA community composition over time, DGGE fingerprinting for thaumarchaeal 16S rRNA genes was performed. DGGE profiles for thaumarchaeal 16S rRNA genes revealed simple patterns and low diversity among different freshwater and marine aquaria (Figure 2). The community composition of the sampled freshwater biofilters was highly similar, with the presence of two or three intense bands shared between different aquaria. The thaumarchaeal 16S rRNA gene patterns associated with the marine aquarium also contained few bands but were distinct from their freshwater counterparts. Dendrograms based on Pearson correlations of DGGE fingerprint densitometric curves placed marine fingerprints in a distinct cluster (Figure 2). NMDS generated from DGGE fingerprints revealed that freshwater and marine aquaria were separated in a two-dimensional space (Figure 3). Consistently, MRPP based on DGGE fingerprints showed significant separation (*T* = −10.83) between freshwater and marine fingerprints. Quantitatively, the distance between different aquaria was assessed by pair-wise ANOSIM. The average distance from the marine aquarium to the freshwater aquaria was significantly higher (0.9, *p*<0.01 as determined by Student's t test) than between freshwater aquaria, indicating that the AOA community in the marine aquarium was distinct from their freshwater counterparts.

**Figure 4:**
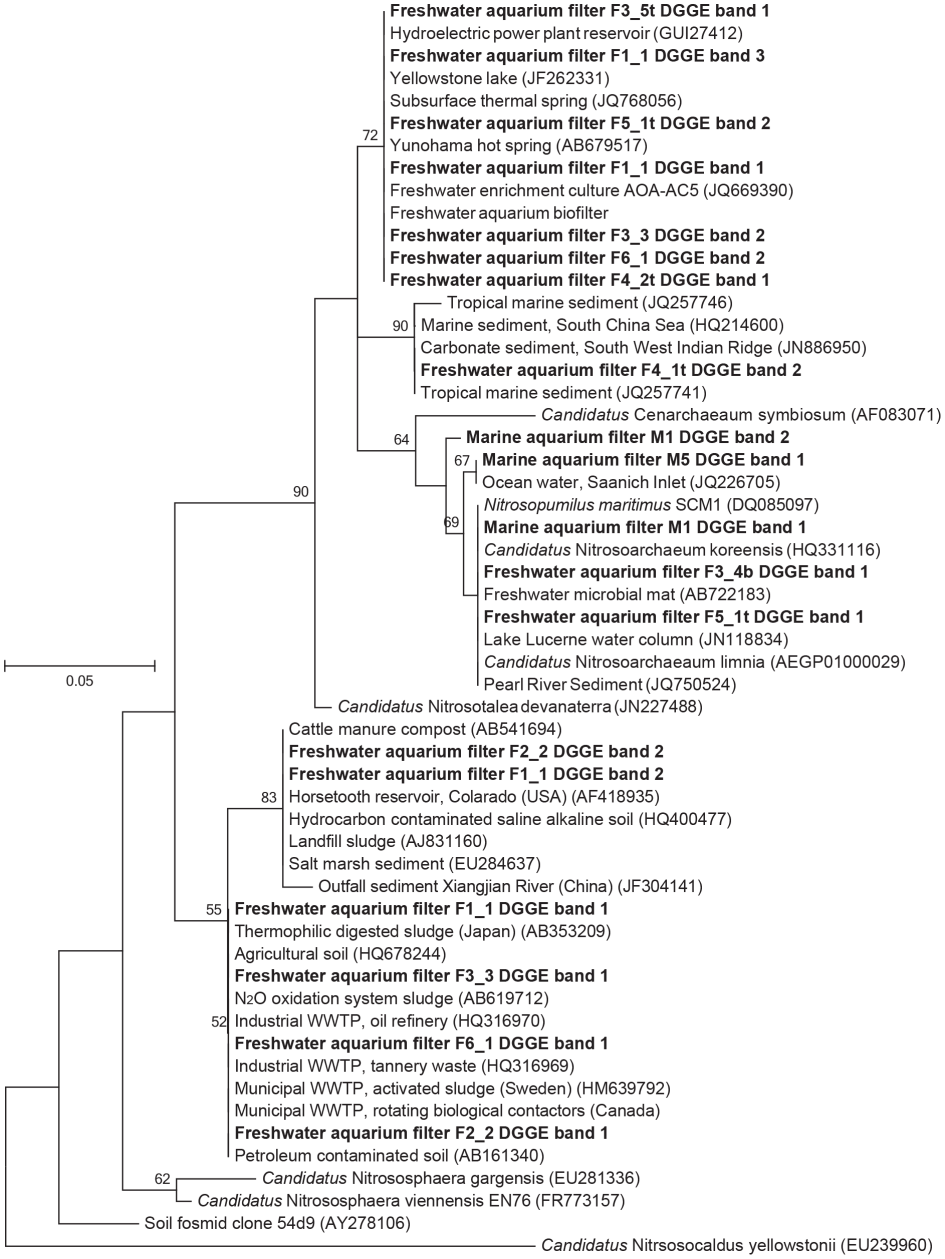
Maximum likelihood phylogenetic tree of thaumarchaeal 16S rRNA gene sequences and DGGE bands based on 500 bootstrap values. Only bootstrap values greater than 50% are indicated. The scale bar represents 5% nucleotide divergence.

We further assessed the temporal dynamics among freshwater AOA communities to determine the stability of the community over time. Freshwater samples were dispersed in the NMDS ordination but there was no gradual succession away from the initial condition (Figure 3). MVDISP showed a higher dispersion for freshwater aquaria F1 and F3 (IMD of 1.37 and 1.24, respectively) by global MVDISP analysis, indicating a change in community from the initial condition of these aquaria. Despite dispersion, communities did not change significantly over time, but instead samples were clustered based by aquarium (*A* = 0.53), with separation (*T* = −11.55) between aquaria as calculated by MRPP. DGGE patterns also revealed similarity in community composition of the sampled freshwater biofilters (Figure 2). Thus, AOA communities in freshwater aquaria were temporally stable despite of their differences in pH (6–8), temperature (19–28°C), dissolved oxygen levels (6.5–9.0 mg O_2_ L^−1^) and housed fish and crustaceans. Representative DGGE bands from freshwater and marine aquaria were sequenced to identify the AOA taxa found within biofilters. Most marine 16S rRNA gene sequences clustered together and shared sequence homology with *N. maritimus.* The freshwater sequences were more diverse and clustered with archaeal sequences from a variety of environments, including saline soils and a wastewater treatment plant (Figure 4).

### Effect of biofilter media type on AOA abundance and diversity

**Figure 5:**
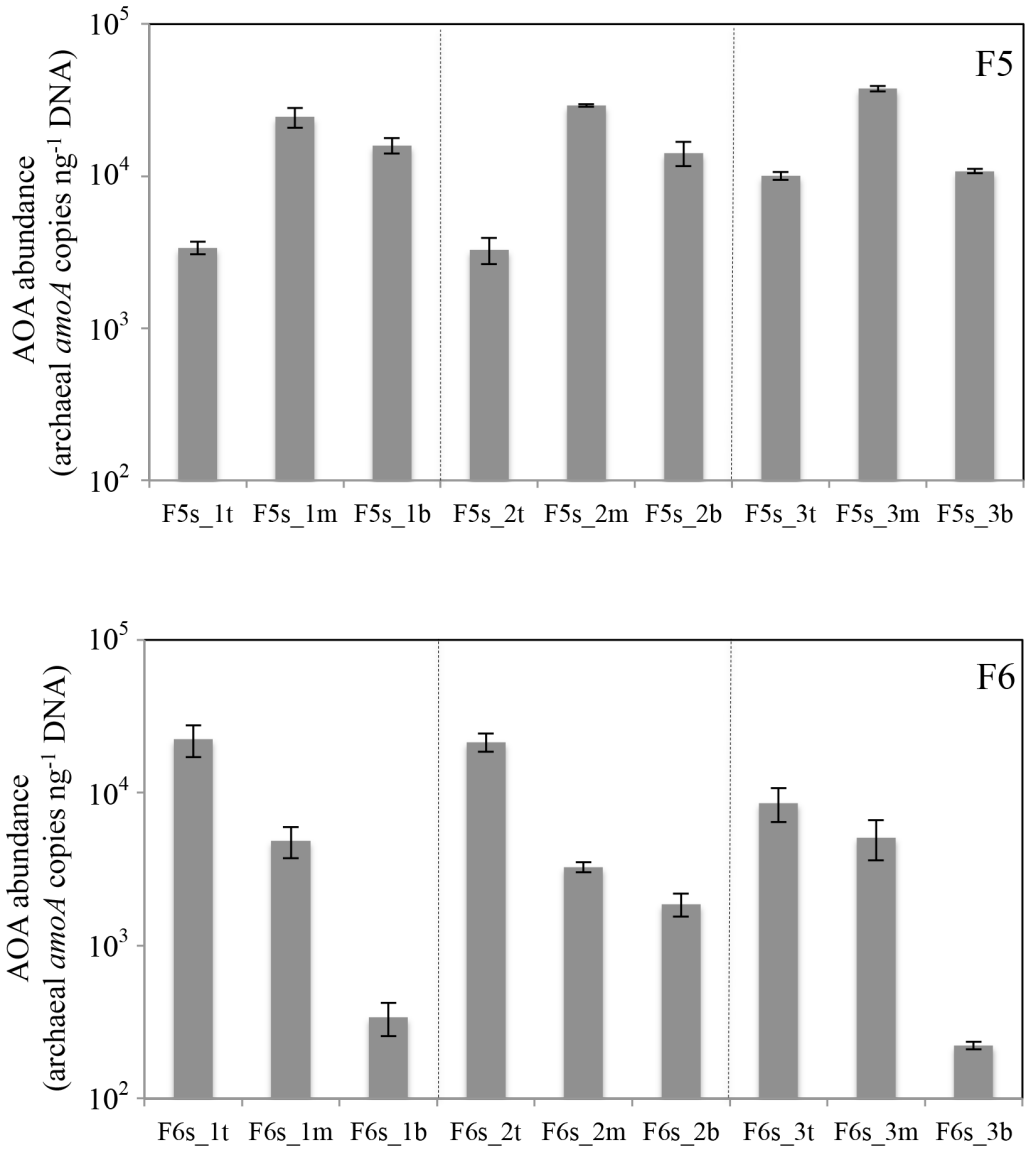
Spatial AOA distribution in multi-media freshwater biofilters F5 (top panel) and F6 (bottom panel). Error bars correspond to standard deviations based on triplicate qPCR amplifications. Dashed lines separate sampling days. For sample labels, refer to Table 1.

**Figure 6:**
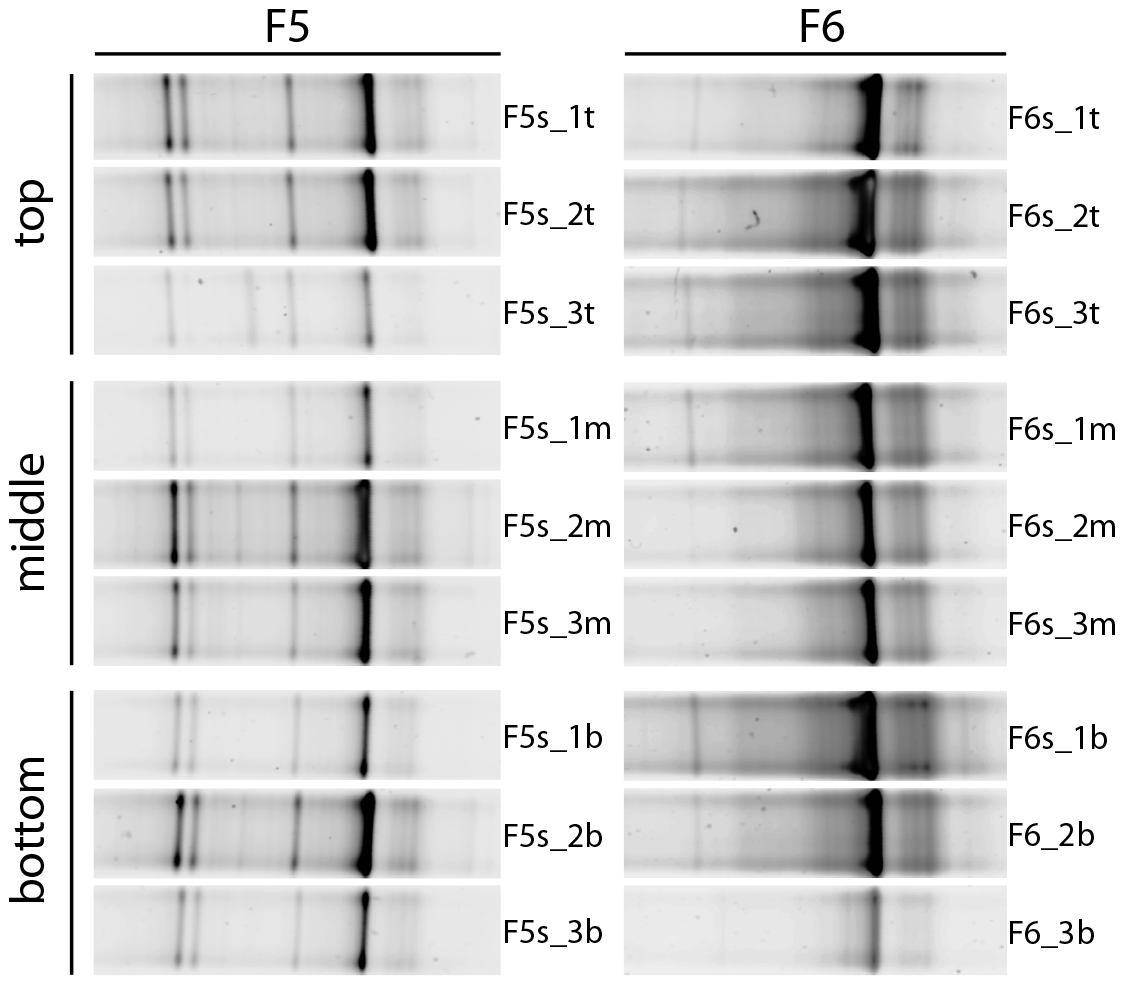
Denaturing gradient gel electrophoresis of thaumarchaeal 16S rRNA genes from spatially distinct locations within freshwater biofilters F5 and F6 during spatial test. For detailed characteristics of the samples, refer to Table 1.

Three-media biofilters of aquaria F5 and F6 were examined to assess the effect of media type on AOA abundance and diversity. In F5, no AOB could be detected, as observed throughout the temporal test period. For F6, a new biofilter was inoculated with biomass originating from the previous F6 biofilter, which had a low AOB abundance. After a start-up and stabilization period of 6 months, AOA had fully outcompeted the AOB community (Table S1). The observed thaumarchaeal *amoA* gene abundances were distinct for the three compartments within each of the two filters, and these trends were consistent over all three sampling time points (Figure 5). For F5, relative AOA abundance was highest in the middle compartment (sponge), followed by the bottom (ceramic) and top (glass) compartments. For F6, relative AOA concentrations peaked in the top compartment (fine sponge), followed by the middle (rough sponge) and bottom (ceramic) compartments. Both filters had a bottom-to-top flow pattern, but the trends in F5 suggest that AOA copy number was not influenced by the position with respect to the water flow. The highest AOA copy numbers were detected in (fine) sponge carrier material for both F5 and F6, suggesting a preferential AOA growth or biofilm attachment to this medium. DGGE profiles for thaumarchaeal 16S rRNA genes revealed low diversity and high spatial stability among different compartments of F5 and F6 aquaria (Figure 6). The community composition of the top, middle and bottom compartment in both F5 and F6 was highly similar with the presence of two or three intense bands. This indicated that although AOA had preferential growth on fine sponge materials, the community composition across the biofilter media types was stable.

### Water chemistry and nitrogen balance

Average TAN concentrations were low and constant in all systems, ranging from <0.1–0.5 mg N L^−1^ (Table 1). Furthermore, nitrite concentrations were always below detection limits (<0.15 mg NO_2_^—^N L^−1^). Hence, all released ammoniacal nitrogen was efficiently and fully nitrified.

Freshwater aquaria F1–F3 and marine aquarium M represented systems without a dedicated nitrogen budget control. Nitrate concentrations varied widely in these aquaria (Figure S1). Nitrate concentrations were <50 mg NO_3_^—^N L^−1^ for F1 and F2, and F3 was characterized by very low nitrate concentrations (∼1.8 mg NO_3_^—^N L^−1^) during initial phase that reached a steady-state nitrate concentration of 0.47±0.04 mg NO_3_^—^N L^−1^ within 60 days. Measured nitrate concentrations fluctuated with water changes in aquarium M; the concentration reached to ∼50 mg NO_3_^—^N L^−1^, but decreased below the detection limit after water change. The range of nitrate concentrations varied from 9–89 mg NO_3_^—^N L^−1^ in aquaria F4-F6, with a decreasing trend over time due to the weekly water changes. Detailed nitrogen budgeting in these aquaria based on nitrogen added as feed, enabled estimation of the minimum biofilter nitrification rates. This approach revealed that 86, 85 and 81% of the nitrogen added through the feed had been converted into nitrate for F4, F5 and F6, respectively, indicating that nitrification is the major nitrogen converting process in the aquarium (Figure S2). Using these conversion percentages, the minimum biofilter nitrification rates were calculated (Table 2). Potential relationships between AOA community composition and water chemistry parameters were assessed with multivariate methods based on relative abundance of thaumarchaeal DGGE bands. Water chemistry parameters and total ammoniacal nitrogen levels had no significant correlation (*R* ^2^>0.3) with AOA community diversity based on DGGE fingerprints despite differences in water temperatures (18.5–28.1°C), pH (6.0–8.0), and DO (6.5–9.1 mg O_2_ L^−1^) (Table 1). Overall, nitrification was the major nitrogen conversion process in the freshwater aquaria where AOA were the dominant ammonia-oxidizing microorganisms.

## Discussion

Our study indicates that AOA are the dominant ammonia-oxidizing microorganisms in freshwater aquarium biofilters, and that their relative abundance and diversity are stable over time. The AOA community of a marine aquarium studied was distinctly different from the freshwater AOA communities, and was accompanied by an AOB community as well. The distribution of AOA within freshwater multi-media filters was stable, and an indication of AOA growing preferentially on fine sponge carrier material was observed by qPCR. Nitrogen balances in freshwater aquaria may suggest that dominant AOA communities oxidized a high proportion (at least 81–86%) of added fish feed nitrogen, demonstrating that nitrogen assimilation by fish or algae or nitrogen removal by denitrifiers played a minor role (maximum 14–19%) of the overall nitrogen budget.

### AOA dominance in freshwater aquaria

Based on a screening of several aquarium biofilters, Sauder and coworkers (2011) first reported qualitative evidence that AOA may act alone in catalyzing ammonia oxidation in freshwater aquaria. Our study further supports the numerical dominance of AOA over AOB in freshwater aquarium, and demonstrated that such dominance was stable over time. Indeed, *amoA* gene abundances indicated that AOA were the sole representative ammonia oxidizers in three out of the six freshwater aquaria tested (Figure 1). A previous study of a drinking water treatment plant also failed to detect any AOB *amoA* genes [32], while others have reported widespread dominance of AOA in freshwater environment [18], [25]. Although AOB appeared in aquaria F1and F6, their abundance rarely exceeded 20% of the total ammonia-oxidizing community. The only exception was aquarium F3, where AOB abundance was high during the initial sampling. Erguder *et al.* [22] suggested that high DO might selectively favour AOB while microaerophilic conditions might represent the preferential niche for AOA [13], [33]. The DO concentration was high in F3 (Table 1), which might have favored AOB growth in the biofilter. The new sponge material placed inside F3 before the start of the experiment might explain AOB dominance during the initial period, because previous research has reported rapid colonization of AOB in newly installed aquarium biofilters [34]. The initial AOB community might have gradually been outcompeted by AOA, under persistent low ammonia concentrations. Indeed, a spatial differentiation of AOB and AOA was observed within one month of operation according to the likely oxygen gradient (AOB in the top filter and AOA in the bottom filter), with a gradual increase in AOA over time (Figure 1).

### Freshwater vs. marine aquarium ammonia-oxidizing community

The abundance of AOB accounted for an average of 41% of detected *amoA* genes in the sampled marine aquarium, and occasionally appeared as the major ammonia-oxidizing community in the marine samples over the study period (Figure 1). Although the analysis of more than one marine aquarium would have been preferred, an AOB *amoA* gene copy number of 2×10^3^ copies ng^−1^ DNA in the marine aquaria was comparably higher than all other tested freshwater aquarium where AOB was detected (Figure 1). Sauder *et al.* [17] reported a similarly high abundance of AOB in marine aquaria relative to freshwater aquaria. Salinity has been reported to be a strong environmental factor in shaping AOB diversity in estuaries [35], [36], indicating its possible role in niche differentiation of AOA/AOB in the tested marine aquarium. However, not all studies are in agreement with such a correlation between AOB abundance and salinity. Bouskill *et al.* [37] has reported the dominance of AOA in more saline, mesotrophic water column of the Chesapeak Bay estuary, but they considered TAN concentration rather than salinity as the cause of elevated AOA community. In this study, TAN concentrations in the marine aquarium were not significantly different than freshwater aquaria (*p*<0.01; Student's t test), and there was no significant correlation between TAN concentration and the AOA community diversity (*R* ^2^>0.3) based on multivariate methods. Further investigation is necessary to identify possible factors responsible for such niche differentiation of AOA and AOB in freshwater and marine aquaria.

### Temporal and spatial stability of AOA communities

An objective of our study was to examine temporal AOA community dynamics and AOA community structure in different compartments of three-media biofilters. We observed high temporal and spatial stability of AOA communities despite diversity among freshwater aquaria water characteristics. Time had little effect on the community diversity in freshwater aquaria and samples were clustered based on aquarium, indicating that aquarium-specific factors likely influenced the specific composition of AOA. Few dominant AOA ecotypes persisted in freshwater aquarium as observed by DGGE patterns. The marine AOA aquarium community clustered distinctly and sequenced bands were closely related to *N. maritimus.* This is consistent with a study by Sakami *et al.* [38], which also reported *N. maritimus* as the dominant AOA in a marine aquarium. Dominance of AOA clones related to *N. maritimus* was also reported in a recirculating aquaculture system for the production of marine shrimp [25].

AOA have been reported to dominate low ammonia environments such as estuarine oxygen minimum zones [9] and the open ocean [12]. Recently, a negative correlation between TAN concentration and AOA abundance was observed in a rotating biological contactor (RBC) flowpath treating municipal wastewater [15] and in freshwater aquaria, AOA were numerically dominant at low TAN concentration [17]. However, our result suggested a preferential AOA niche driven by the support material rather than ammonia concentration. Alves *et al.* [39] recently reported preferential growth of different AOA clades, not solely based on TAN and NO_3_^—^N concentrations, but on an interplay between different physiochemical parameters in an Arctic soil. The fine sponge material has a high surface area that could help to support biofilm attachment, which would be especially advantageous under the high upflow velocities (9.6–19 m/h; Table 2) and short hydraulic retention times (1.2–56 s; Table 2) found in aquarium biofilters.

### AOA and nitrification

TAN and nitrite accumulation was negligible in all aquarium included in this study, indicating rapid and complete nitrification. The discrepancy between fed and nitrified nitrogen (14–19%) can be attributed to a variety of processes, including assimilatory nitrogen uptake by fish, nitrifiers, and heterotrophs. In addition, nitrogen losses could occur due to denitrification, algal or plant growth (minimal given shielding from ambient light), or incomplete hydrolysis of uneaten food and fish faeces. Because AOA accounted for all detectable *amoA* genes in aquaria F2, F4, and F5, and detected AOB *amoA* gene copies were low and inconsistent in other aquaria, it is likely that AOA are responsible for the observed nitrification rates. It remains unclear whether all detected thaumarchaeal *amoA* genes represent nitrifying potential and whether all cells are equally active [14]; nonetheless, changes in *amoA* gene abundances associated with active nitrification in environmental samples provide indirect evidence of relative roles of bacteria and archaea in ammonia oxidation. The complete nitrification in N-balanced aquaria along with the low relative abundance of AOB *amoA* genes indicated a strong relationship between AOA and nitrification in freshwater aquaria. Such predominance of AOA as members of the ammonia-oxidizing community was previously reported for soil [39]–[41] and inside freshwater macrophyte and rhizosphere sediment [30], where AOA outnumbered AOB by 500- to 8000-fold.

### Conclusions and future outlook

Commercial AOB inocula are commonly employed to enhance nitrification in aquarium biofilters. AOB may represent important biofilter colonizers during initial aquarium establishment, as observed in aquarium F3. However, the results of this study suggest that the development and use of AOA supplement may be beneficial for the operational performance of biofilters associated with aquaria or aquaculture systems. The spatial and temporal stability of AOA and low abundance of AOB across aquarium biofilters suggests that further research is required to better understand the activity and dynamics of AOA and AOB in aquarium biofilters, especially during the early aquarium biofilm formation. Overall, this study demonstrates that the numerical dominance of AOA over AOB in freshwater aquarium biofilters is stable over time, and in different media types within the same aquarium. In addition, we have demonstrated low diversity of AOA in aquarium biofilters, which is also temporally stable. Active nitrification in freshwater aquaria has been shown, with at least 81–86% of added nitrogen converted to nitrate, presumably by AOA communities, which are likely the dominant ammonia oxidizers in these biofilters. AOB may play a more substantial role in marine aquaria, and may colonize new biofilters more rapidly than AOA.

## Supporting Information

Figure S1 **Boxplot distribution of nitrate concentrations in aquaria with uncontrolled N balance.** The whiskers represent the upper and lower 25% of the distribution, and asterisks represent outliers.
(TIF)

Figure S2 **Nitrification efficiency of the three well-maintained aquaria with monitored N budget.** The x-axis showing the cumulative nitrogen added as a feed and y-axis represent the cumulative nitrate accumulation from day 56 onwards. Nitrification efficiency was based on linear regression slopes of 0.86, 0.81 and 0.85 for F4-F6, respectively.
(TIF)

Table S1 **Gene copies for AOB** ***amoA*** **and AOA** ***amoA*** **in the sampled aquaria.** T: top of biofilter; M: middle of biofilter; B: bottom of biofilter; BDL: below detectable limit; NA: not applicable.
(DOCX)
