# Supplementary figures and images for: Temporal and Spatial Stability of Ammonia-Oxidizing Archaea and Bacteria in Aquarium Biofilters
Source: PLoS One. 2014 Dec 5;9(12):e113515. doi: 10.1371/journal.pone.0113515 (PMC4257543; doi:10.1371/journal.pone.0113515)

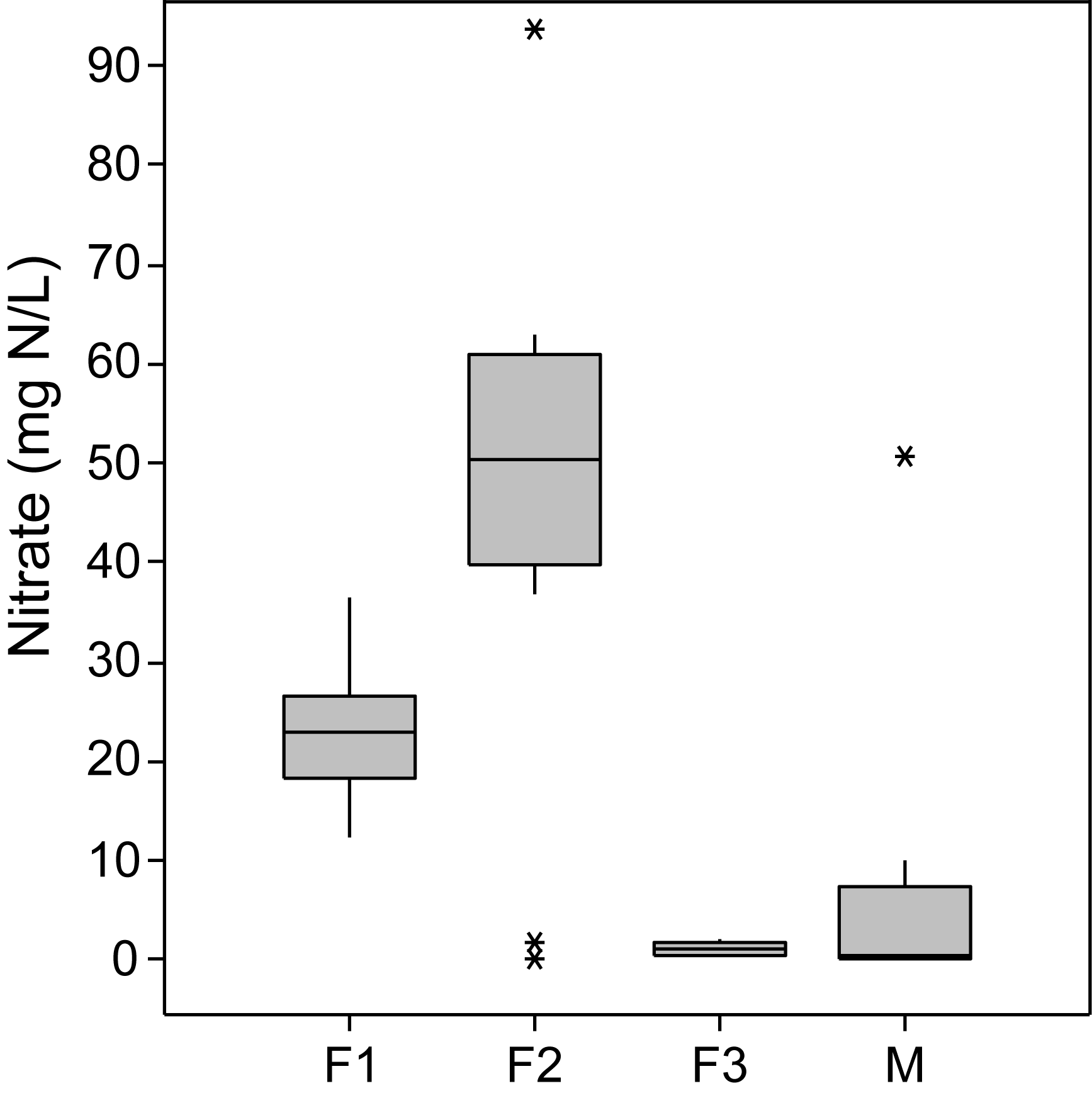

Supplement: Figure S1 — Boxplot distribution of nitrate concentrations in aquaria with uncontrolled N balance. The whiskers represent the upper and lower 25% of the distribution, and asterisks represent outliers. (TIF) [file pone.0113515.s001.tif]

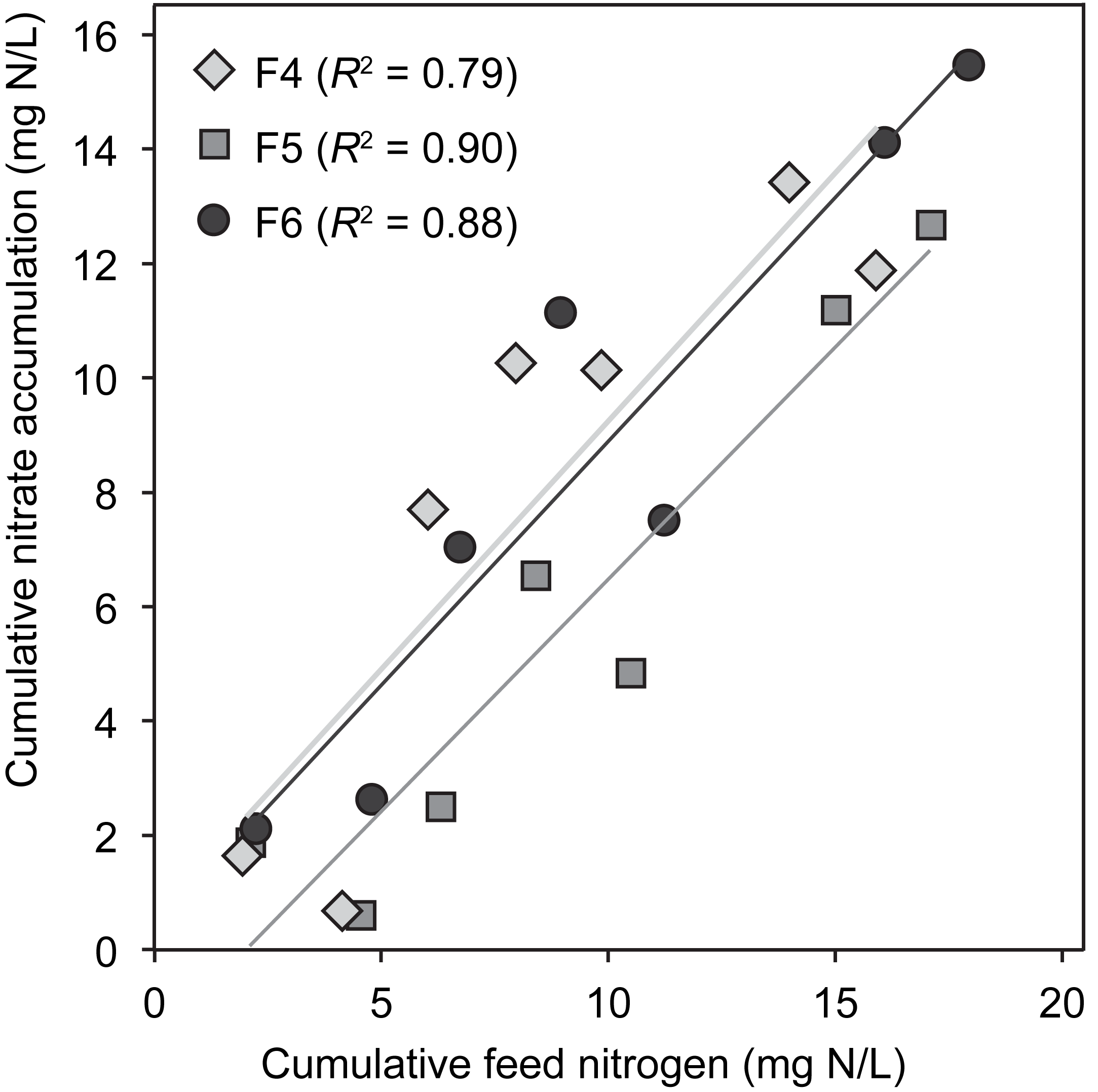

Supplement: Figure S2 — Nitrification efficiency of the three well-maintained aquaria with monitored N budget. The x-axis showing the cumulative nitrogen added as a feed and y-axis represent the cumulative nitrate accumulation from day 56 onwards. Nitrification efficiency was based on linear regression slopes of 0.86, 0.81 and 0.85 for F4-F6, respectively. (TIF) [file pone.0113515.s002.tif]
