# Supplementary material for: Temporal and Spatial Stability of Ammonia-Oxidizing Archaea and Bacteria in Aquarium Biofilters
Source: PLoS One. 2014 Dec 5;9(12):e113515. doi: 10.1371/journal.pone.0113515 (PMC4257543; doi:10.1371/journal.pone.0113515)
Supplement: Table S1 — Gene copies for AOB amoA and AOA amoA in the sampled aquaria. T: top of biofilter; M: middle of biofilter; B: bottom of biofilter; BDL: below detectable limit; NA: not applicable. (DOCX) [file pone.0113515.s003.docx]

**Table S1**. Gene copies for AOB *amoA* and AOA *amoA* in the sampled aquaria. T: top of biofilter; M: middle of biofilter; B: bottom of biofilter; BDL: below detectable limit; NA: not applicable.

| **Aquarium** | **Time (d)** | **Sample** | **DNA (ng/µL)** | **AOA copy no.** | | **AOB copy no.** | |
| --- | --- | --- | --- | --- | --- | --- | --- |
|  |  |  |  | (µL^-1^)^a^ | (µg^-1^ DNA)^b^ | (µL^-1^)^c^ | (µg^-1^ DNA)^d^ |
| F1 | 1 | F1_1 | 76.7 | 468662±107936 | 6110±1407 | 94229±21041 | 1228±274 |
|  | 35 | F1_2 | 83.1 | 11757±538 | 141±6 | BDL | NA |
|  | 55 | F1_3 | 104.7 | 11738±4785 | 136±55 | BDL | NA |
|  | 76 | F1_4 | 82.1 | 94155±11578 | 2401±295 | 68528±17503 | 834±213 |
|  | 104 | F1_5 | 84.9 | 266018±42876 | 4176±673 | BDL | NA |
| F2 | 1 | F2_1 | 39.2 | 1837427±1205219 | 46873±30745 | BDL | NA |
|  | 40 | F2_2 | 63.7 | 58451±2110 | 917±33 | BDL | NA |
|  | 61 | F2_3 | 41.6 | 112618±7564 | 2707±181 | BDL | NA |
|  | 89 | F2_4 | 23 | 30302±5446 | 1317±236 | BDL | NA |
| F3 | 1 | F3_1 | 35.9 | 10147±5553 | 282±154 | 76161±3931 | 2121±109 |
|  | 28 | F3_2t | 27.1 | 7505±2879 | 276±106 | 240312±66738 | 8867±2462 |
|  |  | F3_2b | 22.7 | 21305±1405 | 938±61 | BDL | NA |
|  | 57 | F3_3 | 22.6 | 5676±1915 | 251±84 | BDL | NA |
|  | 85 | F3_4t | 26.9 | 86876±14976 | 3229±556 | BDL | NA |
|  |  | F3_4b | 20.8 | 6815±241 | 327±11 | BDL | NA |
|  | 113 | F3_5t | 18.4 | 191373±26449 | 10400±1437 | BDL | NA |
|  |  | F3_5b | 23.7 | 8725±1711 | 368±72 | BDL | NA |
| F4 | 1 | F4_1t | 117.6 | 156091±36878 | 1327±313 | BDL | NA |
|  |  | F4_1b | 139.7 | 497565±91121 | 3561±652 | BDL | NA |
|  | 22 | F4_2t | 55.3 | 775774±112492 | 14028±2034 | BDL | NA |
|  |  | F4_2b | 44.4 | 65179±5009 | 1468±112 | BDL | NA |
|  | 50 | F4_3 | 61 | 2440890±289134 | 40014±4739 | BDL | NA |
|  | 79 | F4_4 | 37.1 | 997127±80738 | 26876±2176 | BDL | NA |
| F5 (temporal test) | 1 | F5_1t | 54.4 | 164397±11551 | 1397±98 | BDL | NA |
|  |  | F5_1b | 122.7 | 70745±7880 | 506±56 | BDL | NA |
|  | 22 | F5_2 | 36.6 | 26437±4987 | 478±90 | BDL | NA |
|  | 50 | F5_3t | 44.4 | 83672±8813 | 1884±198 | BDL | NA |
|  |  | F5_3b | 39 | 266933±45669 | 6844±1171 | BDL | NA |
|  | 79 | F4_4 | 40.7 | 275178±70517 | 6761±1732 | BDL | NA |
| F5 (spatial test) | 1^e^ | F5s_1t | 35.2 | 118933±11513 | 3379±327 | BDL | NA |
|  |  | F5s_1m | 31.4 | 769749±116036 | 24514±3695 | BDL | NA |
|  |  | F5s_1b | 33.7 | 536371±61563 | 15916±1827 | BDL | NA |
|  | 20^e^ | F5s_2t | 42.3 | 138866±27201 | 3283±643 | BDL | NA |
|  |  | F5s_2m | 71.7 | 2087131±42453 | 29109±592 | BDL | NA |
|  |  | F5s_2b | 43 | 610755±109925 | 14204±2556 | BDL | NA |
|  | 40^e^ | F5s_3t | 42.7 | 429729±26020 | 10064±609 | BDL | NA |
|  |  | F5s_3m | 65.3 | 2460675±103944 | 37683±1592 | BDL | NA |
|  |  | F5s_3b | 48.1 | 519614±16810 | 10803±349 | BDL | NA |
| F6 (temporal test) | 1 | F6_1 | 35.9 | 298733±978 | 8321±27 | 1009±341 | 18±15 |
|  | 29 | F6_2 | 45.6 | 206082±56 | 4519±1 | 1360±102 | 29±2 |
|  | 57 | F6_3 | 80.6 | 140470±5816 | 1742±72 | 7939±523 | 98±6 |
|  | 86 | F6_4 | 43.2 | 1252464±209454 | 28992±4848 | 6500±829 | 150±19 |
| F6 (spatial test) | 1^e^ | F6s_1t | 56.1 | 1257972±297803 | 22424±5308 | BDL | NA |
|  |  | F6s_1m | 71.2 | 346356±80452 | 4865±1130 | BDL | NA |
|  |  | F6s_1b | 40.4 | 13721±3391 | 340±84 | BDL | NA |
|  | 20^e^ | F6s_2t | 63 | 1349627±183246 | 21423±2909 | BDL | NA |
|  |  | F6s_2m | 39.2 | 128127±9865 | 3269±252 | BDL | NA |
|  |  | F6s_2b | 35.1 | 65524±11100 | 1867±316 | BDL | NA |
|  | 40^e^ | F6s_3t | 33.9 | 291217±73293 | 8590±2162 | BDL | NA |
|  |  | F6s_3m | 40.7 | 207514±60971 | 5099±1498 | BDL | NA |
|  |  | F6s_3b | 33.2 | 7386±420 | 222±13 | BDL | NA |
| M | 1 | M_1 | 32.8 | 8600±2895 | 262±88 | 25397±6957 | 774±212 |
|  | 14 | M_2 | 28.9 | 4424±972 | 153±33 | 3931±1053 | 136±36 |
|  | 63 | M_3 | 39.9 | 3008±177 | 75±12 | BDL | NA |
|  | 77 | M_4 | 35.8 | 4084±582 | 114±16 | BDL | NA |
|  | 105 | M_5 | 24.7 | 10084±429 | 408±17 | 50416±9627 | 2041±389 |

^a^minimum detection limit: 213; ^b^ minimum detection limit: 4.2; ^c^ minimum detection limit: 1000; ^d^ minimum detection limit: 9.7; ^e^  Spatial test start day was 6 months after temporal test.
